# Immunotherapy in hepatocellular carcinoma: the complex interface between inflammation, fibrosis, and the immune response

**DOI:** 10.1186/s40425-019-0749-z

**Published:** 2019-10-18

**Authors:** Bridget P. Keenan, Lawrence Fong, Robin K. Kelley

**Affiliations:** 1 Division of Hematology/Oncology, Department of Medicine, University of California San Francisco, Room M1286, 505 Parnassus Ave., San Francisco, CA 94143 USA; 2 Helen Diller Family Comprehensive Cancer Center, University of California San Francisco, San Francisco, CA USA

**Keywords:** Immunotherapy, Hepatocellular carcinoma, Checkpoint blockade, Liver cancer, Immunology, Fibrosis

## Abstract

Hepatocellular carcinoma (HCC) is the third leading cause of cancer deaths worldwide and confers a poor prognosis. Beyond standard systemic therapy with multikinase inhibitors, recent studies demonstrate the potential for robust and durable responses from immune checkpoint inhibition in subsets of HCC patients across disease etiologies. The majority of HCC arises in the context of chronic inflammation and from within a fibrotic liver, with many cases associated with hepatitis virus infections, toxins, and fatty liver disease. Many patients also have concomitant cirrhosis which is associated with both local and systemic immune deficiency. Furthermore, the liver is an immunologic organ in itself, which may enhance or suppress the immune response to cancer arising within it. Here, we explore the immunobiology of the liver from its native state to chronic inflammation, fibrosis, cirrhosis and then to cancer, and summarize how this unique microenvironment may affect the response to immunotherapy.

## Main text

### Introduction

Hepatocellular carcinoma (HCC) is a disease with both a grim prognosis and rising incidence. The most up to date estimates demonstrate a median overall survival of 9 months for all stages of untreated HCC, a number that worsens with increasing Barcelona Clinic Liver Cancer (BCLC) stage [1]. In the world, liver cancer is the third leading cause of cancer mortality while in the United States, it is the fourth highest cause [2, 3]. The rising incidence of liver cancer in the United States is attributed to the epidemics of hepatitis C virus infection and non-alcoholic fatty liver disease [4, 5]. For early stage HCC, standard of care treatments include resection, localized therapies such as ablation and radiation, and liver transplantation [6]. Until recently, the only first line systemic therapy approved for advanced HCC was the anti-angiogenic multikinase inhibitor sorafenib, based upon prolongation of median survival by approximately 3 months with low rates of tumor radiographic response, attributed to a mechanism of disease stabilization [7].

Immunotherapy is a cancer treatment strategy that has been explored for many years but only recently has seen clinical success, mainly in the form of immune checkpoint inhibitors. Antibodies to the immune checkpoints PD-1, CTLA-4, and PD-L1, have proved to be relatively safe and beneficial in treating triple negative breast cancer, renal cell carcinoma, melanoma, urothelial carcinoma, squamous cell carcinomas of the head and neck, Merkel-cell carcinoma, and non-small cell lung cancer, among others [8–14]. Checkpoint inhibition (CPI) blocks the negative regulatory signals either directly on T cells or on cells that interact with T cells (such as tumor cells, stromal cells, and antigen-presenting cells), providing a stimulus to pre-existing anti-tumor immunity. Recently, two PD-1 inhibitory monoclonal antibodies, pembrolizumab and nivolumab, received regulatory approvals in the second-line setting for advanced HCC as monotherapy [15, 16]. There is also early phase clinical trial data demonstrating activity from anti-CTLA-4 inhibition alone and in combination with transcatheter arterial chemoembolization (TACE) or ablation in a subset of patients [17, 18]*.* Response rates range from 10 to 25% among the different checkpoint inhibitors used, and clinical data are reviewed more extensively elsewhere [19, 20]. Moreover, despite the potential concern for relatively worse toxicity related to CPI due to already poor liver function in the HCC population, overall clinical trials have shown an acceptable safety profile for HCC patients, with rates of immune-related toxicity similar to that in patients with other tumor types and without underlying hepatic dysfunction [21, 22].

The site of HCC development, the liver, makes immunotherapy a promising yet complicated strategy for treatment. First, the liver itself is an immune organ, with rich populations of immune cells, some of which are unique to the liver such as Kupffer cells [23]. As there are elements that can promote both tolerance and anti-tumor immunity within the liver, evidence for the use of CPI in HCC must be inferred from model systems and from the clinical data. In other solid tumor types, metastases to the liver portend a poor response to CPI and are associated with decreased tumor infiltration of CD8^+^ T cells, demonstrating the power of the liver to generate tolerance to tumors derived from other sites [24]. Multiple examples from mouse models further substantiate the induction of systemic tolerance when exogenous antigens are expressed in hepatocytes, an effect mediated by T regulatory cells (Tregs) [25, 26]. Conversely, NK and NK T cells are thought to be potent anti-cancer effector cells, of which the liver has a particular abundance [27–29]. Next, upwards of 80–90% of HCC arises in the context of underlying liver injury which can progress to fibrosis or cirrhosis; therefore, it is important to take into account the variable effects on the immune microenvironment in this state of fibrosis and chronic inflammation [30]. Lastly, the toxic and viral insults that promote carcinogenesis in the liver may drive immunosuppression directly through host/viral interactions or via chronic inflammation, although conversely, pathogen-associated molecules could serve as a source of neo-antigens to be recognized by effector T cells [31]. Thus, there is a tightly interwoven, exceedingly complex, relationship of chronic inflammation and the anti-cancer immune response in the liver which may represent an opportunity for CPI in HCC, but also demands thoughtfully designed treatment strategies to subvert suppressive mechanisms.

### Normal liver biology: a complex balance between tolerance and immunity

**Fig. 1:**
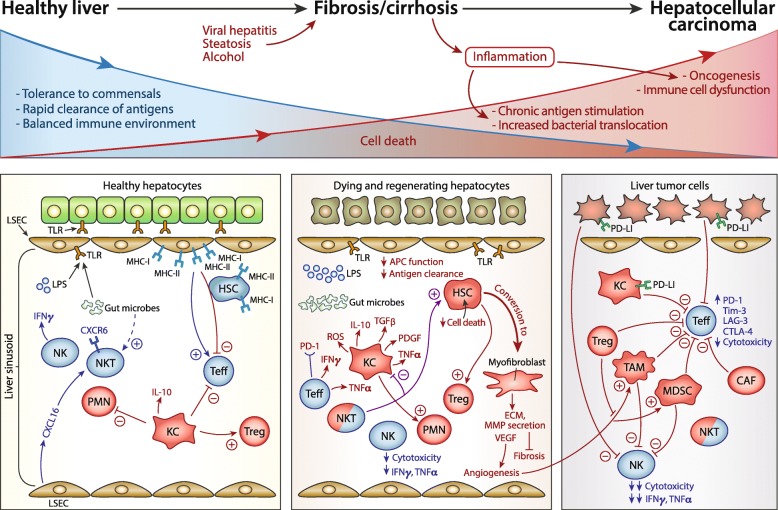
Liver immunobiology across a spectrum from healthy liver to inflammation and oncogenesis. Top panel: Viral and toxic insults drive inflammation in the liver and alter the normal baseline response to gut commensals. Chronic inflammation can lead to alteration of normal immunity to both commensal organisms and pathogens, and eventually, to oncogenesis. Bottom panel: General mechanisms underlying tolerance and immunity and interactions between various cell types are outlined in each of the following states: healthy liver (left), fibrosis and cirrhosis (middle), and hepatocellular carcinoma (right). Cells that generally maintain tolerance in healthy liver and promote immune suppression and oncogenesis are colored in red while cells that favor protective anti-microbial or anti-tumor immunity are colored in blue

The liver is an immune organ made up in bulk by hepatic parenchymal cells. Besides the biliary epithelium, the majority of the remaining 20 % are non-parenchymal cells such as stellate cells, macrophages, NK, and T cells including TCRγδ T cells (Table 1, Fig. 1) [32, 33]. The unique anatomy of the liver puts lymphocytes in direct apposition to hepatocytes through the lack of a basement membrane in liver sinusoids [32]. Due to the chronic antigen load from the gastrointestinal tract, the liver needs to maintain a level of tolerance to balance elimination of gut bacterial pathogens while avoiding severe inflammation induced by non-pathogenic gut commensals. The liver also serves as a major producer of immune-related molecules like C-reactive protein (CRP) and soluble pattern recognition receptors (PRRs) for molecules derived from pathogenic organisms, thus playing a central role in systemic inflammation and immunity [33].

**Table 1:** Immune cell functions and alterations across the spectrum of healthy liver, fibrosis, and hepatocellular carcinoma

Condition						
Cell type	Healthy Liver	References	Fibrosis and chronic inflammation	References	Hepatocellular carcinoma	References
CD8^+^ T cell	Provide protection against infection	[32]	Progressive dysfunction and exhaustion, PD-1 upregulation with chronic inflammation and viral infection	[90]	Anti-tumor antigen-specific responses detected; Progressive dysfunction and exclusion from tumors, upregulated exhaustion markers, low production of granzyme B and perforin, decreased proliferation	[96, 98, 116, 124–126]
CD4^+^ Treg	Antigen-specific tolerance; Readily expand following interaction with HSCs, Kupffer cells, and LSECs	[25, 26, 37]	Secrete IL-10 and TGFβ; Inhibit CD8^+^ T cell responses; Promote B cell activation and production of IgG through CD40-CD40L interaction	[84, 85]	Increased numbers of Tregs found within liver tumors; Suppress CD8^+^ T cell production of perforin and proliferation; Inhibit CD4^+^ effector T cell proliferation; Suppress NK function including cytotoxicity and IFNγ production	[96–98, 107]
CD4^+^ Th cell	Anti-microbial protective immunity; Regulators of pro- and anti-inflammatory signals	[32, 37]	Decreased numbers of naïve CD4^+^ T cells in circulation in cirrhotic patients; Increased numbers of Th17 cells, IL-17 can promote fibrosis via activation of stellate cells	[37, 83, 87, 88]	Elevated CD4/CD8 ratio predictive of recurrence free survival; Increased expression of PD-1 and CTLA-4, Decreased cytokine secretion in intra-tumoral CD4^+^ cells compared to peripheral blood CD4^+^ T cells	[96, 99]
B cell	Not well characterized, few B cells found in healthy liver	[32]	Role not as well-defined; found to be activated in chronic liver disease	[85]	Rarely found via IHC staining of liver tumors, IgA-producing cells suppress CD8^+^ T cells	[94, 113]
TCRγδ T cell	Recognition of peptide and non-peptide ligands; Innate-like and adaptive T cell protection from pathogens	[33]	Production of pro-inflammatory IL-17; Recruitment of CD8^+^ T cells and Th1 cells; Killing of HSCs; Promote monocyte differentiation into MDSCs	[37, 86]	Possible anti-tumor cytotoxicity	[118]
Kupffer cell	Induction of tolerance to commensal bacteria and food particles; Recruit Tregs; Recruitment and clearance of neutrophils; Stimulate T cell response to infection; Recruit and activate NK cells via IL-12 and cell:cell contact	[23, 28, 34–37, 39, 63]	Lose tolerogenic properties under inflammatory conditions; Secrete reactive oxygen species, TGFβ, PDGF, TNFα, and matrix metalloproteinases; Activate HSCs	[23, 33, 61, 65]	Protective against tumors via clearance of tumor cells; Suppression of T cell function via PD-L1 expression	[38, 111]
MAIT cell	Protection against bacteria; React to lipid antigens	[37, 43, 44]	Exhausted phenotype with upregulation of PD-1 and CTLA-4; Capable of activating HSCs	[92, 93]	Potential anti-tumor cytotoxicity; Excluded from tumors and found at higher frequencies in surrounding tissue	[44, 96]
NK cell	Anti-viral protection through cytokine production and cytotoxicity	[28]	Protect against fibrosis by killing of HSCs and production of IFNγ; Can induce liver injury by worsening inflammation	[27, 28, 33]	Cytotoxic to tumor cells; Impaired function (decreased granzyme and perforin, decreased cytotoxicity) and decreased in number in tumors and peripheral blood; Decreased expression of KIR2DL1 and KIR2DL3	[28, 94, 97, 100]
NK T cell	Th1-like phenotype in the presence of IL-12; Th2-like phenotype in the presence of IL-7. Type I NK T cells: Activate neutrophils and HSCs, cause hepatocyte death. Type II NK T cells: Suppress pro-inflammatory signaling pathways.	[28, 45, 46]	Type I NK T cells: Activation of HSCs and neutrophils, production of IFNγ and IL-4 can worsen inflammation	[45, 72]	Type I NK T cells associated with tumor control; Impaired cytotoxicity, decreased expression of KIR2DL1 and KIR2DL3	[71, 100]
Hepatic stellate cell	Express MHC I and II; Induce tolerance and anti-microbial immunity; PD-L1 expression leading to T cell apoptosis	[23, 39]	Differentiate to myofibroblasts; Secrete matrix metalloproteinases, extracellular matrix remodeling; Secrete IL-6, TNFα and TGFβ, Induce Th17 cells and Tregs	[39, 59, 61, 65, 88]	Induce MDSC and polarize monocytes to an immunosuppressive phenotype; Promote tumor growth	[42, 64]
Liver sinusoidal endothelial cell	Expression of MHC I and II; Activate CD4^+^ and CD8^+^ T cell responses; Induce tolerance via PD-L1 expression; Induction of Tregs	[35, 39, 40, 50]	Impaired antigen-processing and lower MHC II expression in the setting of fibrosis related to high levels of circulating endotoxin	[41]	Induce tolerance to tumor-derived antigens; decrease ability of dendritic cells to stimulate T cell responses	[42, 47]
Bone marrow-derived monocyte, macrophage, and dendritic cell	Promote tolerance to commensals and food particles; Stimulate T cell response to infection; More tolerogenic than activating in healthy liver	[39, 47]	Dysfunctional antigen presentation; Increased non-classical monocytes; Production of pro-inflammatory cytokines (TNFα, IL-6, IL-1)	[41, 77, 78, 82, 83]	Conversion to MDSC capable of suppressing effector T cells, inducing Tregs, and promoting tumor growth through pro-angiogenic cytokine production; Conversely, can control tumors via induction of antigen-specific T cell responses; Impaired ability to penetrate tumor tissue	[23, 42, 47, 64, 104]

There are many cell types and molecules involved in maintaining tolerance to gut antigens. Kupffer cells, which are tissue macrophages that develop independent of bone marrow-derived infiltrating monocytes, are located in the lumen of liver sinusoids and are exposed to microbial products, comprising the first line of defense (and tolerance) to pathogens [34]. Kupffer cells are activated by LPS, the complement system, and other pathogen-associated molecular patterns (PAMPs), through the expression of Toll-Like Receptors (TLRs), including TLR2, TLR3, and TLR4, to recognize microbial antigens and signals from damaged hepatocytes [23, 35]. The cytokines produced by Kupffer cells in response to TLR signaling subsequently recruit and activate neutrophils [35, 36]. Neutrophils ingest bacteria, undergo apoptosis following destruction of pathogens, and then are cleared by Kupffer cells which dampens inflammation [36]. Compared to monocyte-derived macrophages, Kupffer cells favor tolerance by expression of IL-10 which induces Tregs and PD-L1 under steady-state conditions [23, 37]. Kupffer cells are also the first line of defense from cancer cells derived from other organs that metastasize to the liver [38]. Further contributing to tolerance, monocytes and dendritic cells (DCs) can be recruited to the liver from the bone marrow; once there, cytokines such as macrophage colony-stimulating factor and hepatocyte growth factor induce a tolerogenic phenotype [39].

Two non-bone marrow-derived cell types unique to the liver, liver sinusoidal endothelial cells (LSECs) and hepatic stellate cells (HSCs), are critical to these interactions with gut flora and mediation of tolerance by the liver. Liver sinusoidal endothelial cells (LSECs) are specialized endothelial cells which sample portal venous blood and act as antigen-presenting cells (APCs) with the ability to cross-prime T cells [40, 41]. LSECs constitutively express TLR4 resulting in NFκB signaling and produce inflammatory cytokines and reactive oxygen species in response to LPS [35, 42]. HSCs are specialized fibroblasts that can transition to myofibroblasts capable of producing extra-cellular matrix proteins which can lead to fibrosis and cirrhosis in some settings, as discussed further below; they can also express MHC I and II and may play a role in T cell priming [39, 42].

Unique innate and innate-like lymphocyte populations exist in the liver, in higher abundance than in other organs or peripheral blood. Natural killer (NK) cells make up 25–40% of hepatic lymphocytes, with important roles in protecting against fibrosis and defending against cancer and viruses through potent cytotoxicity as well as production of IFNγ [28]. Mucosal-associated invariant T cells (MAIT cells) have semi-invariant T cell receptors (TCR) and are capable of mounting an immune response to bacteria [43, 44]. Finally, NK T cells have semi-invariant TCR chains and recognize endogenous and exogenous lipids including those derived from gut microbes. There are two types of NK T cells (I and II) with type II being more numerous in humans; cross-regulation between these cell types is essential for balance of pro and anti-inflammatory pathways in normal liver [45, 46] (Table 1). Although more abundant in the liver than in peripheral blood, NK T cells constitute a relatively small fraction of the total liver T cells and MAIT cells account for a larger portion of the innate-like T cells in humans compared to mice [33, 37].

Conventional T cells must migrate through liver endothelium and, through interaction with APCs mediated by the integrins ICAM-1 and VCAM-1, can be triggered to proliferate upon antigen encounter [37]. The liver contains abundant adaptive and innate-like T cells that protect against pathogens under normal conditions, with higher numbers of CD8^+^ than CD4^+^ T cells, and higher proportions of TCRγδ cells than in the peripheral blood [32, 33, 47]. While Tregs are found at low levels (for example, in comparison to the spleen) at steady state, they are readily induced under tolerogenic conditions by HSCs, LSECs, and Kupffer cells [37, 39]. Effector T cells can be tolerized and clonally deleted following recognition of antigen by direct hepatocyte-induction of apoptosis or apoptosis due to incomplete activation [37].

Beyond cell types and liver anatomy, there are several important tolerance-mediating molecules that have an important role in healthy liver biology. Among them, TGFβ has pleiotropic effects in the liver including promoting fibrosis, carcinogenesis, and hepatocyte death, and during the steady state, is involved in liver regeneration [48]. PD-L1 is constitutively expressed by sinusoidal cells and Kupffer cells, promoting tolerance both under steady state and during viral infection [37, 49]. Additional examples of liver tolerance are documented from the literature on liver transplantation. Given the tolerogenic potential of the liver due to its role in mediating host response to gut flora, perhaps it is not surprising that some liver transplant recipients can fully accept their allograft and safely discontinue immunosuppressive medications [50]. While the full mechanisms for this are not yet fully elucidated, an NK and TCRγδ T cell gene signature identifies patients who establish tolerance of their liver allograft [51]. Tregs are also important in mediating transplant tolerance in mouse models, and Treg cell therapy is an active area of research in the transplant community as a bridge to decreasing or discontinuing immunosuppression post-transplant [52, 53].

### Changes in the liver immune microenvironment with progression from chronic inflammation to fibrosis

Both the systemic and local immune systems, as well as components of innate and adaptive immune systems, are altered in the setting of liver fibrosis and cirrhosis which occurs due to chronic inflammation from toxins, infectious agents, or other insults such as steatosis (Table 1, Fig. 1). It is well-known clinically that cirrhotic patients are systemically immunocompromised and infections constitute a major source of mortality in end-stage liver disease [54]. Bacterial infection and sepsis occur in part due to increased bacterial translocation through a “leaky” gastrointestinal barrier created by portal hypertension, as well as due to weakened systemic and local immunity [54–57]. The exact mechanisms related to the initiation of inflammation from each type of insult are reviewed extensively elsewhere and therefore are not discussed here. Rather, we focus on the general mechanisms involved in fibrosis initiation and changes in the immune status during progression to cirrhosis, an irreversible state that is the end-stage of fibrosis [58].

The main cell types involved in liver fibrosis initiation appear to be HSCs and Kupffer cells. As a result of inflammation due to toxins such as alcohol, steatosis, or viral infection, inflammatory cytokines activate HSCs through TLR4, which then produce extracellular matrix proteins such as collagen [59–61]. The cytokine IL-17 can drive production of pro-fibrogenic IL-6, TNFα and TGFβ by HSCs and Kupffer cells [62]. Mouse models of liver fibrosis demonstrate that under inflammatory conditions, Kupffer cells no longer induce tolerance to experimental antigens as they do in normal liver [63]. During liver damage, Kupffer cells produce reactive oxygen species, TGFβ, and platelet-derived growth factor (PDGF) which activates HSCs [23, 33, 64]. Both Kupffer cells and HSCs secrete matrix metalloproteinases during chronic tissue injury which is mediated by TNFα and TGFβ, promoting remodeling of the extracellular matrix [65, 66]. Fibrosis and the buildup of extracellular matrix leads to a hypoxic environment which results in VEGF upregulation, which may later support tumor angiogenesis [64].

NK cells can protect against fibrosis via killing of activated HSCs, although can also drive inflammation [27, 28]. There is an inverse correlation between IFNγ-producing NKp46^high^ NK cells and the degree of fibrosis in HCV-infected patients [67]. NK cell killing of HSCs and production of IFNγ become suppressed over time with advancing fibrosis and can be further suppressed by alcohol consumption, as seen in a carbon tetrachloride-induced fibrosis mouse model [68]. STAT1 signaling is an important negative regulator of the fibrosis pathway, opposing the effects of TGFβ secreted by HSCs and supporting NK cell cytotoxicity [69]. HSCs become more resistant to NK cell killing in later stages of fibrosis due to SOCS1 upregulation by HSCs [70]. As far as NK T cell populations’ role, there may also be duality based on the particular type of NK T cells involved. Type I NK T cells are thought to be protective in acute liver injury but harmful in chronic inflammation as they activate HSCs and neutrophils whereas in the setting of liver tumors, type I NK T cells are associated with tumor control [45, 71, 72].

As liver injury and fibrosis progress, the extracellular matrix becomes stiffer and the normal anatomy of liver is altered which can then cause impaired production of key immune molecules normally supplied by the liver such as complement pathway proteins. Cirrhotic patients have lower levels of C3 and C4 proteins than healthy controls, associated with infections and mortality, while in contrast and perhaps surprisingly, cirrhotic patients had higher levels of mannan-binding protein and opsonization [73, 74]. Another group that found while mannose-binding lectin (MBL) is not necessarily lower in cirrhotic patients compared to healthy controls, lower levels of MBL in cirrhosis are associated with an increased risk of infections [75]. As fibrosis progresses, a dysfunctional immune response feeds forward the cycle of inflammation. For example, patients with cirrhosis have higher levels of TLR2 expression and circulating endotoxin leading to exaggerated responses to bacterial products [35]. However, the TLR signaling apparatus can become dysfunctional rather than protective against infection, as with more infections seen in cirrhotic patients with TLR4 polymorphisms and with TLR2 and TLR4 dysfunction [55, 76]. Higher circulating levels of endotoxin and IL-10 in cirrhotic patients compared to healthy controls has been associated with “immune paralysis” – an inability of APCs to upregulate MHC and present antigens effectively to T cells [41, 77]. Patients with primary biliary cirrhosis were found to have defective Fc-Receptor mediated clearance of pathogen/antibody complexes, one proposed mechanism of the impaired phagocytosis by APCs that is seen in liver disease, although this has not been seen in alcoholic cirrhotic patients [78]. Low albumin levels in cirrhotic patients can drive neutrophil dysfunction; as albumin binds excess endotoxin, elevated endotoxin levels lead to chronic signaling in innate immune cells as a consequence of hypoalbuminemia [79]. Other pro-inflammatory molecules such as soluble CD163 and MCP-1, activators of macrophages, also are increased in the serum of cirrhotic patients [80, 81].

The chronic high levels of pro-inflammatory chemokines and cytokines alters both systemic and local immune cell subsets from that seen in patients without liver disease. Compared to healthy controls, cirrhotic patients have an increased number of activated monocytes and specifically, more non-classical (CD16^+^) monocytes, which increase with progressive fibrosis and are capable of activating HSCs [82, 83]. Cirrhotic patients with ascites are found to have lower numbers of naïve CD4^+^ and CD8^+^ T cells and higher numbers of activated CD4^+^ T cells in the peripheral blood, as well as increased production of IL-10 and TGFβ by T cells [83, 84]. Suppressive Tregs expressing CD40 ligand occur in both mouse models of liver injury and explanted hepatitis C (HCV)-positive livers [85]. IL-17, capable of activating HSCs and Kupffer cells to produce collagen via activation of the STAT3 pathway, is mainly secreted by T cells, including TCRγδ T cells [62, 86]. Tregs and Th17 cells are both found to be increased in more advanced HBV-related fibrosis compared to earlier stage fibrotic livers; however, an elevated Th17/Treg ratio has been shown to correspond with higher liver stiffness measurement, a correlate of worsening liver fibrosis [87, 88]. Furthermore, chronic antigen stimulation can lead to T cell exhaustion, with upregulation of inhibitory receptors such as PD-1 and progressive loss of polyfunctional cytokine production [89]. Patients with chronic viral hepatitis have exhausted viral-specific T cells; blockade of the PD-1/PD-L1 pathway can partially reverse T cell dysfunction and has demonstrated some success in control of chronic viral infection [90, 91]. In autoimmune liver disease, MAIT cells are also found to be exhausted with less IFNγ production and upregulation of PD-1 and CTLA-4 is seen in autoimmune liver disease and hepatitis B infection [92, 93].

### HCC tumor immunobiology in the fibrotic liver microenvironment

#### Immune cell dysfunction is associated with HCC

HCC often arises in a background of inflamed liver due to toxins and infectious agents, although there are patients in which de novo HCC occurs without known fibrosis and cirrhosis, implying additional pathways to oncogenesis such as viral insertional mutagenesis in the case of hepatitis B virus. However, as the majority of patients that would be potential candidates for immunotherapy have HCC that occurs in the setting of liver fibrosis/cirrhosis, we focus on the immune microenvironment in the context of underlying fibrosis (Fig. 1). Studies of the structural organization of liver tumor versus surrounding non-tumor liver tissue using immunohistochemistry (IHC), and more recently, single cell RNA sequencing, show an immune gradient in the evolution from fibrosis to cirrhosis to cancer. CD8^+^ T cells can penetrate within the HCC microenvironment with surrounding CD4^+^ T cells and B cells, particularly in a subset of lymphocyte-rich tumors; however, in other IHC studies, Tregs are most abundant in central areas with CD8^+^ T cells restricted to borders of tumors [94–96]. Tregs were enriched in the tumors of patients compared to peripheral blood or surrounding tissue adjacent to liver tumor [96]. CD20^+^ B cells and CD56^+^ NK cells were rare via IHC staining of HCC tumors and surrounding liver tissue; in particular, the CD56^low^CD16^+^ NK cell subset, typically characterized by enhanced cytotoxicity, are decreased in peripheral blood of HCC patients versus healthy controls and within tumor versus non-tumor liver, a finding associated with more Tregs [94, 97]. Single cell analysis of immune cells from blood, tumor, and surrounding “normal” liver in HCC patients revealed predominant MAIT cells in non-tumor liver tissue and a high frequency of CTLA-4^high^ Tregs and CD8^+^ T cells with upregulated exhaustion markers in tumor tissue [96]. For the most part, Tregs had unique TCRs suggesting they were not derived from other CD4^+^ T cells, unlike CD8^+^ T cells which had higher degree of overlap in their TCR repertoire between activated and exhausted cells [96].

While CD8^+^ T cells and NK T cells have been shown to be protective against liver tumor cells in mouse models, CD8^+^ TIL found within HCC in patients have been shown to be dysfunctional with low production of granzyme and perforin, low proliferation as measured by Ki-67, and upregulation of exhaustion markers such as TIM3, LAG3, PD-L1, and CTLA-4 [29, 98, 99]. Similarly, NK and NK T cells in tumors of HCC patients were found to have lower expression of KIR2DL1 and KIR2DL3, receptors that modulate NK cytotoxicity, compared to the NK and NK T cells in livers of healthy controls [100]. The dysfunction of effector cells within the tumor microenvironment is driven directly by HCC tumor cells as well as indirectly by suppressive immune cells recruited to tumors. Tumor-associated fibroblasts can suppress NK cell cytotoxicity and cytokine production via signaling intermediates such as prostaglandins and indoleamine 2,3-dioxygenase (IDO) [101]. Soluble MHC class I-related chain A (MICA), an inhibitory NKG2D ligand, secreted by tumor cells, binds to NK cells, thus impairing their ability to activate DCs [102]. Myeloid-derived suppressor cells (MDSC) and tumor-associated macrophages, capable of inducing Tregs and suppressing T cells, are present in HCC mouse models [103] and patients [104]. Angiogenic factors such as VEGF and FGF, are highly expressed by HCC cells and recruit MDSC to tumors [105].

#### An immunosuppressive signaling axis drives progression from chronic inflammation to HCC

Through analysis of paired tumor and non-tumor liver samples from HCC patients, an immunosuppressive gradient has been described with increased expression of chemokine networks such as CXCR3/CXCL10 and CCR6/CCL20 which enhances macrophage and Treg recruitment to the liver [106, 107]. Layilin, a molecule not previously known to be important in HCC and identified with single cell RNA sequencing approaches, is upregulated in CD8^+^ T cells and Tregs and can suppress IFNγ production when over-expressed in CD8^+^ T cells [96]. TGFβ, a driver of liver fibrosis and oncogenesis via induction of hepatocyte apoptosis and subsequent proliferation, can also promote oncogenesis as a key molecule in the induction of Tregs, polarization of macrophages, and suppression of effector T cells [108–110]. PD-L1, expressed by Kupffer cells at baseline in healthy liver, is more highly expressed in areas of tumor compared to normal liver [111, 112].

#### Immune system dysfunction is driven by viral and non-viral insults

While there is likely overlap in the final pathways leading to immune suppression and oncogenesis between the different toxic and infectious insults that lead to HCC, there are also distinct pathways associated with various HCC etiologies. For example, IgA-producing cells in patients with fatty liver disease-related HCC have been implicated in driving oncogenesis via suppression of CD8^+^ T cells [113]. T cells from patients with NASH-related HCC had higher levels of CTLA-4 and OX40, which was also associated with certain serum fatty acid levels; whereas patients with HCV-related HCC had higher numbers of circulating CD45RA^−^ Tregs [114]. A recently published analysis of hepatitis B (HBV)-positive HCC versus non-virally related HCC using mass cytometry and RNA sequencing found several distinguishing characteristics based on etiology. In non-viral HCC, there is generally more IFNγ, IL-17, Granzyme B, and TNFα whereas virally-associated tumors have increased PD-1 expression on T cells, supporting a generally suppressive environment created by HBV [115]. Tregs and CD8^+^ resident memory T cells (TRM) were more abundant in tumors in HBV^+^ patients than HBV^−^ patients and had different transcriptome signatures, such as increased IL-10 signaling pathway in Tregs and more exhaustion-related genes in TRM in HBV^+^ patients [115]. In contrast, TIM-3^+^CD8^+^ T cells and CD244^+^ NK cells were more abundant in the tumors of non-viral HCC [115].

Regardless of initiating injury, impaired liver function leads to alteration of the microbiome and resulting host:microbial interactions and downstream metabolic pathways [56]. Mice treated with antibiotics to deplete gut microbes had less microbial-driven conversion of primary to secondary bile acids which resulted in enhanced CXCL16 expression and recruitment of activated type I CXCR6^+^ NK T cells, protecting against liver tumor growth [71]. Given the effect of liver dysfunction on bacterial translocation and recent studies illuminating the role of the microbiome in response to checkpoint inhibition, there is likely many mechanisms by which the altered gut flora of patients with HCC shapes the immune response within the liver.

### Protective and tumor-antigen-specific immune responses in HCC

Effector cells that are found within tumors and peripheral blood of HCC patients are generally dysfunctional, although existence of certain effector cells and other immune mediators are shown to be associated with improved prognosis, such T and NK cells, suggesting a productive immune response to HCC is possible [116, 117]. TCRγδ T cells are expanded in the blood of liver cancer patients and show capability to kill tumor cells ex vivo [118]. A 14-gene panel of immune-related genes (including TNF, CD8A, IFNG, and various chemokines and TLRs) predicted prognosis in early stage but not late stage HCC, suggesting that a protective immune microenvironment can exist in early but not late stage HCC [119]. CXCL10, CCL5 and CCL2 correlated with infiltration of CD8^+^ T cells, Th1 CD4^+^ T cells, and NK cells [119]. Cytokines such as IFNγ, TNFα, and TLR3 ligands could induce production of these chemokines by cancer cells which then serve to recruit T and NK cells [119]. Myeloid cells can be induced via CpG oligonucleotides to stimulate CD8^+^ T cells, demonstrating the dichotomous nature of myeloid compartment under different conditions [120]. V-domain Ig suppressor of T cell activation (VISTA), while thought to be a negative regulator of T cells, is associated with better prognosis in HCC, in contrast to its association with worse outcomes in other tumor types [121–123]. The association of VISTA with tumor-infiltrating CD8^+^ T cells in HCC may be a signal of activated, albeit exhausted, effector cells that are protective against tumor progression whereas in melanoma and pancreatic cancer, VISTA was mainly expressed by myeloid subsets.

Tumor antigen-specific responses can occur in HCC, and the association of HCC with pathogens such as the hepatitis viruses may be an opportunity for targeting non-host antigens that will be recognized as foreign to the immune system. Spontaneous tumor antigen-specific T cell responses have been detected in HCC, including in a patient that had a complete response following treatment with sorafenib and in another patient cohort following local or systemic chemotherapy [124, 125]. TCR sequencing identified a concentration of shared TCR α and β chains in liver tumors compared to T cells in blood or adjacent non-tumor liver, implying clonal T cells within tumors [96]. Another group found that while there were detectable T cell responses to tumor-associated antigens, responses declined with advancing disease and tumor antigen-specific CD8^+^ T cells were dysfunctional with low production of IFNγ, Granzyme B, and perforin [126]. In a mouse model of HCC driven by virally-induced SV40 large T antigen, there is clearance of most virally-infected cells and in cells that persist, they retain expression of viral products [127]. However, despite the frequency of virally-associated HCC, the response to CPI does not occur in the same high proportion of patients as in other virally-associated cancers such as Merkel cell carcinoma, suggesting that anti-viral immune responses are not sufficient for a successful response to immunotherapy [128].

### Changes in the HCC tumor microenvironment with the use of CPI

Due to limited clinical data overall thus far for the use of CPI in HCC, correlative studies using samples from CPI-treated liver cancer patients has lagged behind that in other cancer types. Therefore, most data we have regarding the changes in the liver post-CPI are derived from mouse models of HCC in which various checkpoint inhibitors have been used. In mouse models of HCC, anti-PD-1 has been shown to have activity both as monotherapy and in combination with other anti-cancer therapies. Due to the heterogeneity of models available, of which none wholly replicates the process of HCC initiation and progression in humans, the results are variable and based on the model used [129]. Importantly, anti-PD-1 has been shown to have activity in mouse models of HCC that incorporate a fibrotic liver microenvironment and that replicate findings seen in human tumors such as progressively exhausted PD-1^+^ CD8^+^ T cells and accumulation of Tregs, as well as in patients with Child Pugh B liver dysfunction [22, 130]. Sorafenib therapy upregulated PD-L1 in orthotopic liver tumors and caused the accumulation of suppressive macrophages and Tregs which could be mitigated with a CXCR4-antagonist [131]. Anti-PD-1 showed synergy with the combination of the CXCR4 antagonist and sorafenib although not with sorafenib alone, demonstrating that a multi-targeted approach may be needed to overcome a suppressive microenvironment [131]. This model is particularly clinically relevant as many HCC patients will have been treated with tyrosine kinase inhibitors prior to CPI which may alter the tumor microenvironment.

To date, the published clinical trials of CPI in HCC have reported relatively limited immune profiling analyses on blood and archival tumor samples in subsets of patients. In the CheckMate040 and KEYNOTE-224 clinical trials of anti-PD-1 therapy, there were no cases of HCV or HBV viral re-activation. In CheckMate040, there were transient decreases in HCV viral load in HCV-infected patients [15, 16]. In patients with HCV and HCC treated with anti-CTLA-4, the majority had a decrease in viral load, including three patients with a transient complete viral response; however, anti-viral T cell responses did not correlate with tumor response [18]. In another study combining anti-CTLA-4 therapy with ablation, anti-viral responses were again seen in HCV^+^ patients and those patients who did not have an anti-viral response also did not benefit in terms of tumor control [17]. This clinical trial included on-treatment biopsies at the time of ablation, which revealed that CD8^+^ T cell infiltration at six weeks after initiation of anti-CTLA-4 correlated with tumor response [17]. In other tumor types, PD-L1 has been used as a predictor of response to anti-PD-1 CPI. In CheckMate040, no association was found between radiographic response and tumor cell expression of PD-L1, whereas the KEYNOTE-224 trial of pembrolizumab, which used a combined score of the tumor and microenvironment immune cell PD-L1 expression, found a correlation between PD-L1-expression and response [15, 16].

### Conclusion

The unique immunobiology of the liver promotes oncogenesis and tumor tolerance under conditions of fibrosis and chronic inflammation, while also presenting an opportunity for therapeutic targeting with immune checkpoint inhibitors. While toxic and pathogenic insults may provide neo-epitopes and pathways to target with anti-cancer agents, the background of chronic inflammation promotes immune suppression in an immune organ already predisposed towards tolerance. Beyond immune cell populations unique to the liver, other factors associated with chronic liver disease may also shape the response to immunotherapy. The microbiome has been demonstrated to predict response to CPI in other malignancies and is particularly relevant to HCC, due to the altered microbiome in the setting of gut translocation in chronic liver disease patients [56, 132]. The microbial contribution, including both gut commensals and pathogenic hepatitis viruses, to oncogenesis and response to CPI should be two key focus areas of future investigation. While mouse models cannot fully recapitulate the complex interaction of fibrosis caused by various toxic and pathogenic insults, the altered liver architecture seen in cirrhosis, and human immune cell populations unique to the liver, several relevant models have thus far demonstrated the benefits to using combination therapies to simultaneously stimulate effector T cells and inhibit suppressive immune populations [130, 131]. Compared to tumor types which are considered immunologically “cold” (having very little immune cell infiltration), the rich infiltrates of leukocytes within the liver present an opportunity to use novel immunotherapy combinatorial strategies to re-polarize immune cells to a productive anti-cancer response. Furthermore, strategies targeting suppressive populations such as HSCs and MDSC that worsen fibrosis and impair protective T cell function are a potential path forward to enhancing the efficacy of CPI. Additionally, tumor cell intrinsic mechanisms of resistance to CPI should be explored. Given that the majority of HCC patients have developed cancer in a background of impaired liver function and liver inflammation, the clinical need for strategies that are both effective and safe in this population is of great importance, as well as determining how best to sequence or combine available agents for HCC. Identification of biomarkers of immune response is also paramount in guiding choice of individual treatments and sequencing therapy. Further correlative and basic science studies should reveal the full potential of the immune system to re-shape the dysfunctional liver tumor microenvironment and overcome the barriers to successful anti-cancer immunotherapy.

## Data Availability

Not applicable

## Abbreviations

AFP: alpha-fetoprotein
APC: antigen-presenting cell
CAF: cancer-associated fibroblast
CD: cluster of differentiation
CPI: checkpoint inhibition
CRP: C-reactive protein
CTLA-4: cytotoxic T-lymphocyte–associated antigen 4
CXCL: chemokine (C-X-C motif) ligand
CXCR: C-X-C motif chemokine receptor
DC: dendritic cell
ECM: extracellular matrix
FGF: fibroblast growth factor
HBV: hepatitis B virus
HCC: hepatocellular carcinoma
HCV: hepatitis C virus
HSC: hepatic stellate cell
ICAM-1: intercellular adhesion molecule 1
IDO: indoleamine 2,3-dioxygenase
IFNγ: interferon gamma
IHC: immunohistochemistry
IL: interleukin
KC: Kupffer cell
KIR: killer-cell immunoglobulin-like receptor
LAG3: lymphocyte-activation gene 3
LPS: lipopolysaccharide
LSEC: liver sinusoidal endothelial cell
MAGE-A1: melanoma-associated gene-A1
MAIT: mucosal-associated invariant T cell
MBL: mannose-binding lectin
MCP-1: monocyte chemoattractant protein-1
MDSC: myeloid-derived suppressor cell
MHC: major histocompatibility complex
MICA: MHC class I-related chain A
MMP: matrix metalloproteinase
NASH: non-alcoholic steatohepatitis
NFκB: nuclear factor kappa B
NK T: natural killer T cell
NK: natural killer cell
NKG2D: natural killer group 2D
NY-ESO-1: New York-esophageal squamous cell carcinoma-1
PAMP: pathogen-associated molecular patterns
PD-1: programmed cell death protein 1
PDGF: platelet-derived growth factor
PD-L1: programmed death-ligand 1
PMN: polymorphonuclear leukocyte (neutrophil)
PRR: pattern recognition receptor
ROS: reactive oxygen species
SOCS1: suppressor of cytokine signaling 1
STAT1: signal transducer and activator of transcription 1
TACE: transcatheter arterial chemoembolization
TAM: tumor-associated macrophage
TCR: T cell receptor
TCRγδ: TCR gamma delta
Teff: T effector cell (representing CD8^+^ and CD4^+^ T effector cells)
TGFβ: transforming growth factor beta
Th1: T helper 1
Th17: T helper 17
TIM3: T-cell immunoglobulin and mucin-domain containing-3
TLR: toll-Like receptor
TNFα: tumor necrosis factor alpha
Treg: T regulatory cell
TRM: resident memory T cell
VCAM-1: vascular cell adhesion molecule 1
VEGF: vascular endothelial growth factor
VISTA: V-domain Ig suppressor of T cell activation
